# The impact of a small-group educational intervention for allied health professionals to enhance evidence-based practice: mixed methods evaluation

**DOI:** 10.1186/s12909-019-1567-1

**Published:** 2019-05-06

**Authors:** Sharon Mickan, Joanne Hilder, Rachel Wenke, Rae Thomas

**Affiliations:** 1 Allied Health Clinical Governance, Education and Research, Gold Coast Health, Executive Office Level 4, Block A, 1 Hospital Boulevard Southport, Gold Coast, QLD 4215 Australia; 2 School of Allied Health Sciences, Griffith University, Gold Coast, QLD Australia; 3 Centre for Research in Evidence-Based Practice, Faculty of Health Sciences and Medicine, Bond University, Gold Coast, QLD Australia

**Keywords:** Evidence-based practice, Education intervention, CREATE: classification rubric for evidence-based practice tools in education mixed methods evaluation, Self-efficacy

## Abstract

**Background:**

Healthcare professionals are recommended to use evidence-based practice (EBP) principles to update and improve clinical practice. Well-designed educational initiatives, together with practice and feedback opportunities can improve individuals’ EBP knowledge, skills and attitudes.

**Methods:**

A concurrent mixed methods assessment was designed to evaluate the effectiveness and feasibility of four monthly workshops on allied health professionals’ knowledge, skills, self-efficacy and behaviour. In between workshops, professionals were encouraged to practice and integrate EBP learnings with colleagues in their workplace.

Participants completed three pre and post intervention assessments: Evidence-based Practice Confidence Scale; adapted Fresno test; and an adapted EBP Implementation Scale. A purpose designed satisfaction questionnaire was completed immediately after the educational intervention and follow up focus groups were conducted after 3 months.

Mean change in assessment data was quantitatively assessed and comments from the clinician satisfaction questionnaire and focus groups were thematically analysed and interpreted together with quantitative data using the Classification Rubric for EBP Assessment tools in Education (CREATE).

**Results:**

Sixteen allied health professionals participated in the EBP workshops and completed all baseline and post intervention assessments. Seven clinicians participated in follow up focus groups. All clinicians reported a positive reaction to the learning experience, preferring short monthly workshops to a full day session. They self-reported improvements in self-efficacy (mean change 15 *p* < 0.001) and implementing EBP behaviours (mean change 7, p < 0.001) from pre- to post-intervention. Although the positive change in EBP knowledge measured by the adapted Fresno test was not statistically significant (mean change 10, *p* = 0.21), clinicians described examples of improved knowledge and skills across all five key steps of EBP during the focus groups. A further, post hoc analysis of individual questions in the two self-reported scales indicated consistent improvement across key EBP knowledge and skills.

**Conclusions:**

A tailored small group EBP education intervention can enhance AHPs’ self-efficacy to develop answerable questions, search the literature, critically appraise, apply and evaluate research evidence. Through practicing these behaviours and sharing new learning with their peers, allied health professionals can enhance their capability and motivation to use research evidence to potentially improve clinical practice.

**Electronic supplementary material:**

The online version of this article (10.1186/s12909-019-1567-1) contains supplementary material, which is available to authorized users.

## Background

The implementation of evidence-based practice (EBP) by healthcare professionals is widely recommended as a strategy to integrate clinical expertise with the best available research evidence [1]. Allied health professionals (AHPs) generally understand the importance of using EBP to maintain high quality clinical practice [2]. However, from current evaluation studies there is no one best EBP educational intervention to ensure allied health professionals integrate research evidence in their clinical practice [2–5]. A recent systematic review concluded that well designed EBP training can significantly influence knowledge, skills and attitudes of AHPs [3]. However, the impact of education on behaviour change is less clear [4].

EBP education programmes for AHPs have generally been delivered in intensive bursts between three hours and two days, using a combination of workshops and applied learning [3–5]. While more intensive learning (shorter massed practice) may lead to better short-term acquisition of knowledge, distributed models over longer time periods may result in better longer term retention [6, 7]. In regards to the design of EBP interventions, a recent overview of systematic reviews suggested while there was no significant difference between multifaceted and single component interventions for changing healthcare professionals’ behaviour, the authors recommended critical consideration of the design and implementation of interventions for specific clinical settings [8]. They concluded that it is important to design and tailor interventions to address identified barriers and enhance enablers of the behaviour/s that are the focus of change. Further, it has been suggested that continued practice in looking for, appraising and summarising best evidence and feedback about changes in knowledge, skills and confidence can maintain healthcare professionals’ improved EBP knowledge skills and attitudes over time [4, 9]. In addition, the concept of self-efficacy is considered important for sustained behaviour change [10]. Self-efficacy theory predicts that highly efficacious individuals will choose to participate in learning activities more often, will expend more effort, and persist longer in the face of difficulty than their peers [11].

In a recent study designed to promote physical therapists’ use of research evidence to inform clinical practice, a two-day workshop was followed by five months of small group work focussed on reviewing and synthesising literature around a common clinical interest [12]. Significant improvements in EBP related self-efficacy and self-reported EBP behaviours were found, with the small group collaborative learning considered an active ingredient for success [13]. Long term improvements in EBP self-efficacy and self-reported behaviours were maintained for six months without any active intervention, suggesting the importance of improved self-efficacy in maintaining behaviour change [14].

From 2015, a group of self-nominated EBP champions were identified and supported within a large Australian tertiary non-metropolitan health service, to share knowledge and encourage the use of EBP in clinical practice within their teams. These EBP champions requested further education to increase their knowledge and gain practical strategies to more effectively undertake this role. A mixed methods study was designed to determine the effectiveness and feasibility of tailored small-group EBP educational workshops for these busy AHPs. Current EBP interventions do not typically provide strategies for participants to share their new EBP knowledge and skills with other team members. However, sharing new knowledge may reinforce participants’ confidence and build self-efficacy [15]. Therefore, development of an EBP intervention that incorporates strategies to promote sharing of learning with peers is also indicated.

## Method

### Aim of study

A tailored education programme was developed to address clinicians’ learning needs, utilising small group work across four monthly workshops. We conducted a mixed methods study to evaluate the impact of this EBP education programme on allied health EBP champions’ self-efficacy, knowledge, skills and behaviour. Specifically, we aimed to determine whether small-group EBP educational workshops conducted once a month for four months for clinicians:

- increased their self-efficacy, knowledge and skills,
- increased their self-reported EBP behaviour,
- are feasible to attend, and
- enabled them to integrate new learnings about EBP

### Study design and setting

A concurrent mixed methods evaluation was designed to address the research aims. Pre- and post-intervention quantitative data and post-intervention qualitative data were independently analysed to evaluate EBP knowledge, skill, self-efficacy, behaviour and acceptability. Comparisons were made between qualitative and quantitative data to deepen understanding of the intervention’s impact. Further, qualitative data about perceived facilitators and barriers were sought to understand factors that promoted and hindered participants’ ability to share new learnings with their work colleagues within their own clinical environments.

### Study participants

A request was sent to all eight professional directors of allied health in one health service, inviting them to nominate at least two interested AHPs, who then completed a written expression of interest to participate in the EBP educational intervention. To be eligible to participate, individuals had to be able to attend all four education sessions, and be willing to share learnings via short clinically based action tasks within their clinical teams. Twenty-four AHPs expressed an interest in attending the workshop. Purposive sampling was used to allocate sixteen places, with a maximum of two staff from eight different professional disciplines and representation from different practice settings. Where more than two AHPs from the same practice setting and profession were nominated, two authors (RT and RW) evaluated individual responses on their expression of interest to determine the most suitable.

### Tailored EBP educational intervention

Four monthly two hour workshops were conducted using course material developed by the Centre for Research in Evidence-Based Practice at Bond University, Queensland, Australia (material available upon request). All sessions were facilitated by two EBP academic and research staff (RT and RW) within teaching rooms at one hospital, with eight staff allocated per facilitator. The staff allocated to groups were evenly distributed in regard to gender and profession and both groups used the same educational materials. Short informal didactic teaching was interspersed with group activities and group discussion was supported by a practical workbook that participants were encouraged to complete. Topics addressed the five steps of EBP, and the content for each session closely followed the curriculum for teaching EBP identified in a recently published Delphi survey of health professionals [16]. Practical examples were integrated to encourage allied health EBP champions to use research evidence to inform their clinical practice. For example, the first workshop addressed the first of five steps of EBP and involved teaching participants to formulate an answerable question followed by practical workbook based exercises on matching study designs with different clinical questions. The second workshop addressed the second and third steps of EBP and involved demonstration and application of strategies to search the evidence using participant’s clinical questions, and critically appraise primary and secondary research. The third workshop continued to address the third and fourth steps of EBP and involved teaching and workbook based exercises about interpreting inferential statistics including statistical significance, odds ratios, risk ratios and confidence intervals to assist with critical appraisal and applying evidence in practice. The final workshop addressed the fourth and fifth steps of EBP and focused on applying and evaluating evidence to practice, teaching statistical versus clinical significance and using clinically-based exercises to apply this knowledge. Participants were asked to complete an action task after each workshop, by identifying one learning from the workshop and sharing their new learnings with at least one other colleague during their clinical practice. Participants discussed this action task at the start of the next workshop including a self-reflection of what worked well and what they learnt from the experience of sharing their learning.

### Measurement tools used

Participants completed three standardised assessments immediately before and after participating in the small group educational intervention. Self-efficacy toward EBP was assessed using the Evidence-based Practice Confidence (EPIC) Scale [14]. EPIC consists of 9 items each scored in 10% increments from 0 to 100% confident. Responses were averaged to generate a mean confidence score. This scale has demonstrated excellent test-retest reliability and acceptable construct validity amongst physical therapists [15] and occupational therapists [17].

We assessed EBP knowledge and skills using the adapted Fresno test. This test has demonstrated reliability and content and construct validity for AHPs [18]. Participants chose from two clinical scenarios and answered seven open-ended questions related to the scenario. Responses were graded by an independent assessor, using a standardised scoring rubric. Total scores range between 0 and 156, with higher scores indicating better knowledge and skills. The assessor was blinded to whether the responses were from pre- or post-assessments.

Self-reported EBP behaviours were assessed using an adapted version of the EBP Implementation Scale [19]. The full EBP Implementation Scale has established face, content and construct validity and internal reliability [20]. Of the 18 original items, 13 items were chosen that reflected the content of the workshops. Consistent with an earlier study, participants were not asked to collect, analyse, present, or react to patient data, and the corresponding 5 items addressing these behaviours (5, 7, 15–17) were excluded to avoid masking any observable changes in self-reported behaviour [13]. This scale measures the frequency of specific behaviours, associated with the five steps of EBP. Participants reported how often they demonstrated specific behaviours across an eight week period on a likert scale ranging from 0 (not at all) to five (more than eight times). Responses were summed, with a maximum score of 65, where higher scores indicated greater frequency of EBP implementation.

We assessed feasibility, utility, and acceptability of the EBP workshops immediately post-intervention using a tailored satisfaction questionnaire (Additional file 1). Participants rated their level of satisfaction with the educational intervention on a five point likert scale across five questions. Participants also provided feedback about the workshop across four open ended questions.

Participants were invited to attend a face-to-face semi-structured follow up focus group (3–4 participants, 1 interviewer) within three months of completing the educational intervention. It was facilitated by an independent evaluator not involved in the delivery of the training to reduce potential positivity bias. A focus group template was designed using the seven categories of the Classification Rubric for EBP Assessment Tools in Education (CREATE) framework to investigate perceptions of change in knowledge, skills and self-efficacy (see Additional file 2). Participants were asked to describe examples of changes in their behaviour, decision making and clinical practice. They were also asked for examples of what knowledge and skills they had shared with their clinical teams.

### Data analysis

Change in standardised quantitative assessment data was assessed using paired two-tailed t-tests for normally distributed data and Wilcoxon matched pairs for data that did not follow a normal distribution. When the normality assumption was met, parametric tests were used for likert scales within the EPIC Scale, assuming underlying continuous concepts in each. Alpha was set at 0.05 and 95% confidence intervals were calculated.

Comments from the clinician satisfaction questionnaire were documented and explored to gain insight into the participant’s experiences of the educational intervention, and for examples of how they had integrated their learning with clinical practice and shared knowledge within their teams. Text from all interviews was coded into categories and sub-categories to provide a descriptive summary of the data.

Qualitative and quantitative data were analysed independently and in parallel. The CREATE framework was used as a guiding theoretical framework [19]. Five of the seven categories which represent a developmental progression for educational evaluation were aligned to the original research aims and with the chosen quantitative and qualitative tools (see Table 1). This framework guided comparisons between both data sets, for a deeper level of meaning and interpretation.

**Table 1:** Data Analysis Framework

Research Aims	CREATE Framework	Qualitative Tools	Quantitative Assessment Tools
Feasibility for busy clinicians	1. Reaction to educational experience	Satisfaction Questionnaire (Additional file 1) Follow up Focus Group	
Clinicians’ self-efficacy, knowledge and skills	3. Self-Efficacy	Satisfaction Questionnaire Follow up Focus Group	EPIC Scale
	4. Knowledge		Modified Fresno Test
	5.Skills		
Clinicians’ self-reported EBP behaviour	6. Behaviours		EBP Implementation Scale
Integration of new learnings about EBP	7.Benefits to patients	Follow up Focus Group	

Ethical approval was received from Gold Coast Human Research Ethics Committee (HREC/16/QGC/228).

## Results

Sixteen AHPs participated in the EBP educational intervention. Participants were predominately female (*n* = 12) and aged between 20 and 39 years (*n* = 13) (see Table 2). Pairs of participants represented six different allied health professions including dietetics, medical imaging, occupational therapy, pharmacy, social work and speech pathology, and were accompanied by three physiotherapists and one music therapist. All AHPs worked across a variety of acute and community practice settings. Experience varied from less than two years to more than 15 years with 63% (*n* = 10) having between 5 and 10 years of clinical experience.

**Table 2:** Participant characteristics

Participant details (*n* = 16)	% (n=)
Male	25% (4)
Female	75% (12)
Research higher degree	44% (7)
Attended other EBP course in 2016	12.5% (2)
Years Clinical Experience	
< 2 years	6% (1)
2 - < 5 years	19% (3)
5–10 years	63% (10)
10–15 years	6% (1)
> 15 years	6% (1)

All participants completed baseline and post-intervention assessments. Attendance across the workshops was high, with average attendance being three out of four sessions. All participants were invited and seven participated in the follow up focus groups, three months after the final workshop. This included participants from dietetics (n = 1), occupational therapy (*n* = 2) physiotherapy (n = 1) speech pathology (n = 2) and social work (n = 1). Nine participants declined because of competing work priorities or changing work positions.

Participants’ qualitative and quantitative responses are reported and aligned to the CREATE framework which has been used to focus the interpretation of both qualitative and quantitative comments. Reference will be made to the original research aims, as presented in Table 1.

### Reaction to education experience

**Fig. 1:**
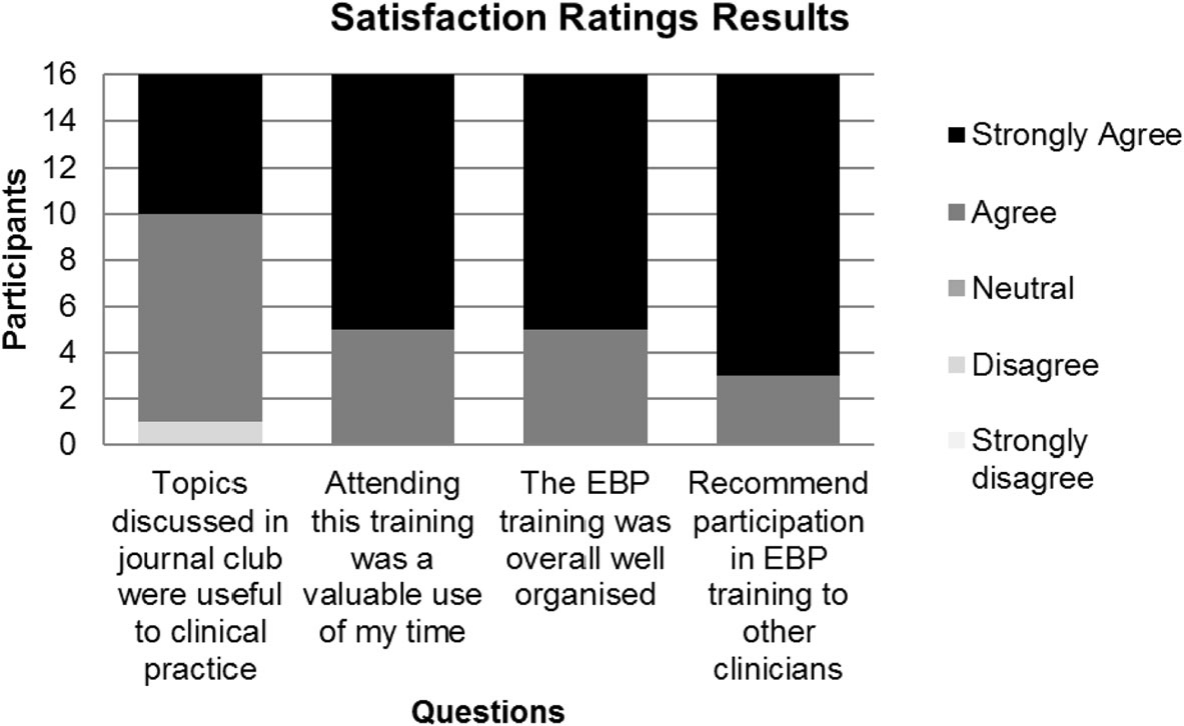
Satisfaction rating results

Overall, likert scale ratings and responses to open questions on the satisfaction questionnaire were positive, supporting the original research aim of feasibility and acceptability for busy clinicians. Fifteen participants (93%) agreed or strongly agreed that topics discussed were useful to their clinical practice. All participants agreed or strongly agreed that the small group EBP intervention was a valuable use of their time, and was well organised. They all reported that they would recommend the small group educational intervention to other allied health professionals (see Fig. 1).

Participants also provided overall positive qualitative feedback about the workshop through focus groups and the satisfaction questionnaire. The workbook was highly valued for both its content: “*the workbook contains both information for reference and exercises/examples to enhance understanding*” (anonymous survey respondent) and as a resource that participants could use after the workshop: “*I loved the resource. I have used that a lot*” (participant 11).

Participants preferred the short monthly sessions: “*I think having eight hours spread over four months was better than one full day*” (participant 13) as it allowed them to fit education into their clinical work: *“I think that workshops held once a month are feasible with our … work load*” (participant 16). The small group size resulted in participants feeling able to discuss the content freely: “*I like how that content was delivered in a room [where] we had about half a dozen people and I was free to speak in an informal way*” (participant 10).

Participants valued the qualified and experienced facilitators who delivered the workshop: “*enthusiastic and knowledgeable presenters with a real world clinical background*” (anonymous survey respondent). Some participants recognised that their enthusiasm to attend the workshop was a reflection of their motivation to learn: “*we all self-nominated to go to this course so we obviously had some motivation to try and improve*” (participant 3).

Participants reported a number of individual barriers to the workshops including complicated subject matter, and their own confidence, as well as time to participate in the workshop: *“it was difficult to take two hours out of a busy clinical day to attend*” (anonymous survey respondent). However, they also recommended that the workshop be run again: “*I really got a lot out of doing the program myself so it would be great to have some other colleagues to be able to have that opportunity as well*” (participant 8). One participant suggested that workshops could be focussed on building the skills of AHPs to teach each other: “*it might be nice if we could work on a train the trainer model… that would be a more realistic way of getting the information out there*” (participant 13).

### Changes in self –efficacy, knowledge and skills, and behaviour

Participants’ quantitative changes in self-efficacy, knowledge and skills, and behaviour are summarised in Table 3. The original aims of increasing participants’ self-efficacy and self-reported EBP behaviours are quantitatively supported.

### Self efficacy

On average, participants improved in their self-rated efficacy from pre- to post-intervention (mean change 15; *p* < 0.001; see Table 3). This increase in self-efficacy is mirrored in participants’ focus group responses. Participants reported feeling more confident: *“I definitely feel more confident that I can look for it in the research*” (participant 16), and more skilled: “*able to find more relevant articles that what I was previously able to find*” (participant 8). Participants also described how their increased confidence encouraged continued practice: “*I’m more confident with the literature now that I have a better chance of being able to build my search strategy in a good way and being able to appraise the information that I get…it’s more worthwhile putting the effort in*” (participant 5).

**Table 3:** Quantitative changes in Self-Efficacy, Knowledge and Skills, and Behaviour

Domain	Assessment	Mean pre (% of max score)	Mean post (% of max score)	Mean difference CI 95%	*p*-value
Self-Efficacy	EPIC Scale TOTAL score (maximum score = 100%)	62 (62%)	77 (77.0%)	15 (7.5 to 23.1)	0.00**
Knowledge & Skills	Adapted Fresno TOTAL score (maximum score = 168)	107 (64%)	116 (69%)	10 (6.3 to 25.5)	0.21
EBP Behaviour	EBP implementation scale TOTAL score (maximum score = 65)	23 (36%)	30 (46%)	7 (3.2 to 9.9)	0.00**

** = statistically significant (*p* = < 0.05)

Given the consistency noted between independent quantitative and qualitative analyses, post hoc tests were conducted for individual items on the EPIC scale. These revealed significant self-reported improvements in all but one of the individual EPIC items (see Table 4). This suggests that individuals were reporting changes in all five steps of EBP, despite starting with different levels of confidence across all abilities.

**Table 4:** Pre and post mean scores for individual items in the EPIC Scale (*n* = 16)

Evidence-based Practice Confidence (EPIC) Scale				
How confident are you in your ability to:	Mean pre	Mean post	Mean difference CI 95%	Sig.
Identify a gap in your knowledge	78	79	1.3 (6.4 to 9.1)	0.78
Formulate a question	66	82	16.2 (11.5 to 20.9)	0.00**
Conduct an online search	61	82	20.6 (12.0 to 29.2)	0.00**
Critically appraise study methods	56	73	17.1 (8.2 to 26.1)	0.00**
Interpret study results	33	64	31.2 (24.0 to 38.5)	0.00**
Determine if evidence applies to patient	63	81	18.1 (11.6 to 24.6)	0.00**
Ask your patient about preferences	72	82	9.4 (0.1 to 18.6)	0.04**
Decide on a course of action	64	77	12.5 (4.6 to 20.4)	0.00**
Evaluate your course of action	62	77	15.0 (5.2 to 24.7)	0.00**

** statistically significant (*p* < 0.05)

### Knowledge and skills

On average, participants reported a positive change in their EBP knowledge and skills as measured by the total score of the modified Fresno test, after they had completed the small group educational intervention, however this was not statistically significant (mean change 10; *p* = 0.21; Table 3). Further, the changes reported on individual items in the EPIC Scale (Table 4) suggest participants were able to recognise improvements in key EBP knowledge and skills. Specific changes in conducting an online search and critically appraising study methods were also described during the focus groups three months after the final workshop: “*being able to find some articles that are relevant to what I’m looking for… as well as reading and understanding the research that was done”* (participant 16). Participants also recognised the clinical relevance of their skills in formulating a question, in terms of an “*increase in [my] ability to turn a clinical problem into a question that I can then research in a structured way…it cuts out some of the time that it takes to investigate something I am interested in*” (participant 8). Participants linked these improvements with feelings of increased confidence: “*I think reading research, comparing relative risk and confidence intervals have definitely become more ingrained and I feel more confident in reading them*” (participant 16).

### Behaviour

Participants self-reported significant improvements in the total score on the EBP implementation scale following the small group educational intervention (mean change; *p* < 0.001; see Table 3). Behaviour changes were consistently described during the focus groups, three months after the educational intervention. Participants described a range of practical changes, including; adapting the structure of a journal club to include critical appraisal tools and independently accessing research: *“rather than referring to my supervisor, [I am] actually going back to the research and doing that* [*literature search] together”* (participant 11). Specifically, they described the consequence of their increased confidence and skills in comments of: *“I am able to find some articles that are relevant to what I’m looking for more easily”* (participant 8) and *“Yeah, I definitely feel more confident…especially in running searches …and helping other staff”* (participant 16). Some participants have actually begun to do their own research: “s*ince doing the training … I’ve started to try and progresses in some research relevant to what the [occupational therapists] are doing out here”* (participant 3).

Given the consistency noted in independent quantitative and qualitative analyses, post hoc tests were conducted for individual items on the EBP Implementation scale. Post-hoc analyses identified that six of the 13 items showed significant improvements following the educational intervention (see Table 5). These included: informally discussing research evidence, generating a PICO question, critically appraising a research study, accessing the Cochrane database and sharing evidence with colleagues. These changes are also consistent with those reported on individual items of the EPIC Scale (Table 4).

**Table 5:** Pre and post mean scores in the EBP Implementation Scale (*n* = 16)

EBP Implementation Scale				
Question: How often have you…	Mean pre	Mean post	Mean Difference CI 95%	Sig.
Use evidence to change my practice?	2.3	2.5	0.2 (0.2 to 0.6)	0.31
Informally discussed evidence from a research study?	2.6	3.2	0.6 (0.0 to 1.2)	0.04**
Shared evidence from a research study with a patient/family?	1.9	2.4	0.5 (0.0 to 1.0)	0.05
Shared a clinical/EBP guideline with colleagues?	2.2	2.4	0.2 (− 0.5 to 0.7)	0.72
Read and critically appraised a clinical research study?	2.2	2.7	0.5 (− 0.2 to 1.2)	0.16
Critically appraised evidence from a research study?	1.7	2.4	0.7 (0.1 to 1.3)	0.02**
Used a clinical/EBP guideline or systematic review to change practice?	1.6	1.5	−0.1 (− 0.4 to 0.5)	0.76
Evaluated the outcomes of a practice change?	1.3	1.4	0.1 (−0.3 to 0.5)	0.48
Shared research evidence with a multi-disciplinary team member?	1.7	2.7	1.0 (0.5 to 1.5)	0.00**
Accessed the Cochrane database of systematic reviews?	1.6	2.1	0.5 (0.1 to 0.9)	0.02**
Shared evidence from a study to more than 2 colleagues	1.4	2.3	0.9 (0.5 to 1.4)	0.00**
Generated a PICO question about my clinical practice?	1.2	2.2	1.0(0.4 to 1.6)	0.00**
Accessed national guidelines?	1.8	2.2	0.4 (0.0 to 0.7)	0.05

** statistically significant (*p* < 0.05)

### Integration of new learnings

In addition to the behaviour changes reported, all focus group participants were able to identify times where they had shared new learnings with their co-workers with many noting the workbook was an enabling tool. They described referring back to the workbook with colleagues: “*when people have had questions we have used that workbook and the skills from the EBP program”* (participant 11). Participants also described demonstrating and sharing practical skills of searching and critical appraisal with their colleagues, specifically: “*helping other staff to run searches and develop PICO [Population, Intervention, Comparator, Outcome] questions”* (participants 11, 16). Participants were seen as a resource person by their peers and they reported: “conducting *in-services with other staff”* (participants 8, 10), and “*sharing summaries with their team members”* (participant 13).

Participants identified specific enablers and barriers to integrating their new learnings and using specific EBP knowledge and skills more broadly in their workplace. Two main enablers were identified. Management support was identified as enabling individuals to access training, work with allied health research fellows and build collaborative relationships with universities: “*if you have got a supportive team or colleagues you can work around … and access experts or other people interested in the area”* (participant 3). Participants also described an emerging research culture that supported clinicians to use EBP in clinical practice: *“I think we have come a long way… but I think it’s acknowledging that there have been some changes and it has made a difference in terms of how services are structured and how we base our practice”* (participant 13).

In addition, participants recognised certain barriers to implementing EBP into practice. Time was the most commonly reported barrier: *“with clinical waitlists and the sheer number of patients that we see in any one day [it is difficult to] …find time in between them”* (participant 6). Other barriers included limited access to allied health journals through the hospital library and limited published evidence in participants’ clinical interest areas.

### Benefit to patients

Participants described the impact for their patients in that they discussed research with them with more confidence: *“I feel when I am talking to my clients about recommendations and what we need to do, comments that we make that are backed up with research carry more weight than what I was doing before”* (participant 6). They also described being more likely to check for research evidence before they intervened with patients: *“I was asked recently about …what the OT role could be in a heart failure exercise programme … rather than just coming up with [something] of our own we are taking a more structured approach [to] look at what [research evidence] is already out there…and it has been beneficial for everyone to go through that process. It feels like you are doing it properly”* (participant 3).

Participants reported that their patients were more receptive to treatment, when they discussed research supporting the treatment with more confidence: *“the clients are a lot more receptive to treatment when you start talking to them in ways like that”* (participant 6).

## Discussion

This mixed method evaluation supports positive change across multiple levels of educational evaluation. Our data suggests that a tailored monthly small group EBP education intervention was feasible for busy AHP clinicians, and it increased self-reported self-efficacy and supported behaviour change in evidence-based practice. All clinicians reported a positive reaction to the monthly workshops in terms of regular attendance with high levels of satisfaction. They self-reported improvements in self-efficacy (mean change 15; *p* < 0.001) and implementing EBP behaviours (mean change 7; p < 0.001) from pre- to post-intervention. Clinicians reported positive change in EBP knowledge measured by the adapted Fresno test, however this was not statistically significant (mean change 10; *p* = 0.215). They also described examples of improved EBP knowledge and skills. Most notably, clinicians self-reported quantitative and qualitative change across all of the traditional five steps of EBP; developing an answerable question, searching the literature, critically appraising the evidence, applying this into clinical practice and evaluating their practice. Finally, clinicians described integrating their new learnings about EBP through sharing knowledge and skills with their work colleagues. In addition, they suggested seeing positive benefits for their patients.

The self-reported quantitative and qualitative improvements in self-efficacy and behaviour change are largely consistent with those reported following the six month Physical therapist-driven Education for Actionable Knowledge translation (PEAK) program [13]. In this program, it was proposed that enhanced self-efficacy enabled physical therapists to continue to use their EBP skills and knowledge to enhance their clinical practice [14]. Further, the long term sustainability of these improvements in EBP knowledge and skills was attributed to therapists’ progressive increased confidence in, and use of EBP to inform their clinical practice.

In the current study, we condensed the actual time of the educational intervention and facilitated the integration of clinicians’ EBP knowledge and skills by explicitly requiring them to share learnings with work colleagues. To make opportunities most salient, we gave clinicians freedom to choose what topic they wanted to share and how they wanted to share it. Sharing opportunities subsequently varied from demonstrating how to run a literature search with peers or students, to running formal in-services to their department. Three months after the intervention, clinicians described continuing to share their practical skills of searching and critical appraisal with their work colleagues.

It seems that an educational intervention focused on building the traditional five steps of EBP can boost self-efficacy and skills quite quickly when there is time and opportunity to practice and improve skills. If there are opportunities to continue to practice new skills in clinically relevant situations, then behaviours can also change. Further insights may be gained from analysis of the key requirements of behaviour change; individual capability, motivation and external opportunities [21]. A tailored small group education intervention can enhance clinicians’ EBP capability and motivation through small group learning that involves practicing skills within clinical scenarios with a self-selected group of enthusiastic learners. Reported increases in behaviours of developing answerable questions, searching the literature and critically appraising the research evidence may be a result of clinicians being more confident to practice and share skills of searching and critical appraisal. In this study, we encouraged clinicians to continue sharing new learnings with peers, but we could not directly support this behaviour change. Despite this, consistent behavioural changes were reported at a three month follow up. Further, these behaviours appear to be important precursors to actually applying evidence to improve clinical practice. So while this initial behaviour change may be observed during and after an educational intervention, it may be that health professionals require more time and practice to achieve objective quantitative improvements in EBP knowledge. Additional consideration of external opportunities such as having management support and sufficient time, and working within a positive research culture may be required to facilitate behaviour change that sustains clinical practice improvements and achieves patient benefits. An example might include active participation in a clinically based journal club. Previously, journal clubs have demonstrated evidence to maintain and enhance EBP skills and behaviours in allied health professionals [22, 23]. The opportunity for sustained behaviour change that supports patient benefit is more complex and needs careful consideration when planning EBP educational interventions. This would require an organisational intervention that identified barriers and readiness for change, and monitored and evaluated outcomes, as suggested by the knowledge to action cycle [24]. Future research should take a longer term follow up of behavioural and knowledge changes after educational interventions, and seek to link them with observable patient changes.

## Limitation and research implications

A key limitation of this study was the small number of highly motivated clinicians in one local health service. Quantitative changes before and after the educational intervention were only realised on the two scales requiring clinicians to self–report. No significant change was observed on the objective measure of EBP knowledge and skill, but this may not be a realistic expectation in such a short time frame. Further, a larger sample of clinicians may be required to detect significant change using this tool [25]. Development of sensitive assessment tools are also required to measure knowledge change and patient benefits.

## Conclusion

A tailored small group EBP educational intervention can enhance AHPs’ self-efficacy to develop answerable questions, search the literature, critically appraise, apply and evaluate research evidence. With opportunities to practice new skills and incentives to share knowledge with peers, clinicians can enhance their capability and motivation to use research evidence to review and improve clinical practice. While four workshops were feasible for busy clinicians to attend and were preferred to a one-day workshop, more time and specific strategies may be required to establish sustained knowledge change and translate to patient benefits. Education is therefore an important pre-cursor for behaviour change, but additional opportunities are required to sustain behaviour change to achieve patient benefits.

## Additional files

Additional file 1: Satisfaction Questionnaire. (DOC 29 kb)

Additional file 2: Follow up Focus Grip Questionnaire. (DOC 25 kb)

## Abbreviations

AHP: Allied Health Professional
AHPs: Allied Health Professionals
CREATE: Classification Rubric for Evidence-based practice Tools in Education
EBP: Evidence-based practice
EPIC Scale: Evidence-based Practice Confidence Scale
HREC: Human Research Ethics Committee
OTs: Occupational Therapists
PEAK Program: Physical therapist driven Education for Actionable Knowledge translation program
PICO: Population, Intervention, Comparator, Outcome – this is a widely used strategy to frame a research question and to develop a search strategy
