# Supplementary material for: The impact of a small-group educational intervention for allied health professionals to enhance evidence-based practice: mixed methods evaluation
Source: BMC Med Educ. 2019 May 6;19:131. doi: 10.1186/s12909-019-1567-1 (PMC6503357; doi:10.1186/s12909-019-1567-1)
Supplement: Supplementary file 1 — Satisfaction Questionnaire. (DOC 29 kb) [file 12909_2019_1567_MOESM1_ESM.doc]

**Additional file 1: Satisfaction Questionnaire**

Have you taken part in any other EBP education in 2016?

YES NO

If yes, please indicate the name of the course and the duration of this training (in hours)

___________________________________________________

Please rate your level of agreement with the following statements in regards to the EBP training you have participated in over the last 4 months:

*I found the topics discussed in the EBP workshop were useful to my clinical practice*

1______________2______________3________________4________________5

Strongly Disagree Neutral Agree Strongly

Disagree Agree

*I think attending this workshop was a valuable use of my time*

1______________2______________3________________4________________5

Strongly Disagree Neutral Agree Strongly

Disagree Agree

*The EBP education format was overall well organised*

1______________2______________3________________4________________5

Strongly Disagree Neutral Agree Strongly

Disagree Agree

*I have shared what I learnt from the workshop with my colleagues*

1______________2______________3________________4________________5

Strongly Disagree Neutral Agree Strongly

Disagree Agree

*Overall, I would recommend participation in EBP education to other clinicians*

1______________2______________3________________4________________5

Strongly Disagree Neutral Agree Strongly

Disagree Agree

What did you find most useful about the EBP workshops you participated in over the last 4 months?

What factors (if any) do you believe helped to facilitate the effectiveness of the workshops?

What (if any) barriers did you encounter which you believe hindered the effectiveness of the workshops?

How do you think the EBP education format that you participated in over the last 4 months could be improved in the future?
