# Supplementary material for: The impact of a small-group educational intervention for allied health professionals to enhance evidence-based practice: mixed methods evaluation
Source: BMC Med Educ. 2019 May 6;19:131. doi: 10.1186/s12909-019-1567-1 (PMC6503357; doi:10.1186/s12909-019-1567-1)
Supplement: Supplementary file 2 — Follow up Focus Grip Questionnaire. (DOC 25 kb) [file 12909_2019_1567_MOESM2_ESM.doc]

**Additional file 2: Follow Up Focus Group Questions**

How would you describe your experience with participating in the EBP training?

How did you find the format of the training?

*(e.g., timing of it, small group format, tasks to share learning with others)*

Have any of your knowledge or skills changed since receiving the training? If so, provide examples

Were there been any examples where you have shared your knowledge with members of your team during the training?

and now since the training?

*(can you give examples?)*

Have there been any changes to your clinical decision making since attending the training? If so, provide examples…

Have there been any other changes to your behaviour since the training? If so, provide examples

*Prompt e.g., are you looking at evidence more, doing searches your self*

Have you been able to integrate what you have learnt into your clinical practice?

What has helped you to do this? *(e.g. networking with other learners, increased confidence?)*

Are there any specific barriers or obstacles to integrating your learning into clinical practice?

Are there any ways that you think these could be addressed?

Moving forward, what do you think could be done to further build EBP within allied health?

How do you think allied health EBP champions could be used in the future?

Is there anything else, you want from research positions or the organisation?

Is there anything you wanted to add that hasn’t already been discussed in relation to your participation in the EBP training programme or EBP in allied health?
